# The East Asian Winter Monsoon Acts as a Major Selective Factor in the Intraspecific Differentiation of Drought-Tolerant *Nitraria tangutorum* in Northwest China

**DOI:** 10.3390/plants9091100

**Published:** 2020-08-27

**Authors:** Hengxia Yin, Lirong Wang, Yong Shi, Chaoju Qian, Huakun Zhou, Wenying Wang, Xiao-Fei Ma, Lam-Son Phan Tran, Benyin Zhang

**Affiliations:** 1 State Key Laboratory of Plateau Ecology and Agriculture, Qinghai University, Xining 810016, China; hengxiayin@qhu.edu.cn; 2 College of Ecological Environment and Resources, Qinghai Nationalities University, Xining 810007, China; 2018002@qhmu.edu.cn; 3 Key Laboratory of Plant Resources Conservation and Sustainable Utilization, South China Botanical Garden, Chinese Academy of Sciences, Guangzhou 510650, China; shiyong@scbg.ac.cn; 4 Key Laboratory of Stress Physiology and Ecology in Cold and Arid Regions, Gansu Province, Department of Ecology and Agriculture Research, Cold and Arid Regions Environmental and Engineering Research Institute, Chinese Academy of Sciences, Lanzhou 730000, China; chaojuqian@lzb.ac.cn (C.Q.); maxiaofei@lzb.ac.cn (X.-F.M.); 5 The Key Laboratory of Restoration Ecology in Cold Region of Qinghai Province, Northwest Institute of Plateau Biology, Chinese Academy of Science, Xining 810008, China; hkzhou@nwipb.cas.cn; 6 Department of Life Sciences, Qinghai Normal University, Xining 810008, China; wenyingwang@qhnu.edu.cn; 7 Institute of Research and Development, Duy Tan University, 03 Quang Trung, Da Nang 550000, Vietnam; 8 Stress Adaptation Research Unit, RIKEN Center for Sustainable Resource Science, 1-7-19 22, Suehiro-cho, Tsurumi, Yokohama 230-0045, Japan; 9 College of Eco-Environmental Engineering, Qinghai University, Xining 810016, China

**Keywords:** *Nitraria tangutorum*, genetic structure, intraspecific divergence, ecological niche modeling, East Asian winter monsoon

## Abstract

The influence of Quaternary climate fluctuation on the geographical structure and genetic diversity of species distributed in the regions of the Qinghai–Tibet Plateau (QTP) has been well established. However, the underlying role of the East Asian monsoon system (EAMS) in shaping the genetic structure of the population and the demography of plants located in the arid northwest of China has not been explored. In the present study, *Nitraria tangutorum*, a drought-tolerant desert shrub that is distributed in the EAMS zone and has substantial ecological and economic value, was profiled to better understand the influence of EAMS evolution on its biogeographical patterns and demographic history. Thus, the phylogeographical structure and historical dynamics of this plant species were elucidated using its five chloroplast DNA (cpDNA) fragments. Hierarchical structure analysis revealed three distinct, divergent lineages: West, East-A, and East-B. The molecular dating was carried out using a Bayesian approach to estimate the time of intraspecies divergence. Notably, the eastern region, which included East-A and East-B lineages, was revealed to be the original center of distribution and was characterized by a high level of genetic diversity, with the intraspecific divergence time dated to be around 2.53 million years ago (Ma). These findings, combined with the data obtained by ecological niche modeling analysis, indicated that the East lineages have undergone population expansion and differentiation, which were closely correlated with the development of the EAMS, especially the East Asian winter monsoon (EAWM). The West lineage appears to have originated from the migration of *N. tangutorum* across the Hexi corridor at around 1.85 Ma, and subsequent colonization of the western region. These results suggest that the EAWM accelerated the population expansion of *N. tangutorum* and subsequent intraspecific differentiation. These findings collectively provide new information on the impact of the evolution of the EAMS on intraspecific diversification and population demography of drought-tolerant plant species in northwest China.

## 1. Introduction

Geological events and climate fluctuations play a major role in shaping the distribution of plant species and their genetic diversity worldwide [1,2]. A more comprehensive understanding of species distribution patterns and population dynamics within those patterns in responses to geological and climate events can provide critical information on climate evolution and the relationship between plant species and the environment. Several studies have attempted to elucidate the influence of the Qinghai-Tibet Plateau (QTP) uplift on diversification of species located in or near the northern edge of the QTP [3,4,5,6]. Some phylogeographical studies have indicated that increased levels of aridification and desert expansion triggered by the QTP uplift are prominent factors driving the speciation and diversification of desert plants [2,3,7,8]. The northwestern part of China, which includes the regions of QTP positioned in the arid regions of the central Asia, has experienced complicate orogenesis that is widely recognized to be closely related with genetic diversification, accelerated variation, and speciation [8,9,10,11].

The impact of climate oscillations, especially climate fluctuations during the Pleistocene, on species distribution patterns has attracted increasing research interest due to the predominant effects of climatic factors on plant adaptation to local habitats [12,13]. Furthermore, the glaciation cycles with arid/cold glacial and humid/warm inter-glacial episodes, might act as more predominant drivers for changes of phylogeographic patterns of the regional vegetation located in northwest China, which has been exemplified in recent studies [14,15,16,17]. This influence is partially plausible in a small time-scale, because multifaceted lines of evidence suggested that the QTP appeared to be barely uplifted since the mid-Eocene and has partly reached over 4000 m above the sea level [18]. In addition, Quaternary glaciation alternations might induce the habitat fragmentation and finally lead to the inter- or intra-specific diversification [14,16]. Thus, under extreme weather conditions during the glaciation periods, distributional ranges of most species in high mountainous or plateau regions were contracted when they migrated into the so-called glacial refugia, and experienced expansion again in postglacial time, thereby leading to the species differentiation or secondary contact evolution [2,10,17,19]. Some investigations have suggested that the locations of glacial refugia for plants were determined mainly by the adaptability of species to the external environment, especially the climatic factors [20,21,22].

As a main component of climatic factors, the monsoon system, especially the East Asian monsoon system (EAMS), which consists of the East Asian summer monsoon (EASM) and the East Asian winter monsoon (EAWM) with humid/warm and dry/cold characters, respectively [22,23], may play crucial roles in affecting the genetic structure of plant species in northwest China. More specifically, the drier and colder EAWM may more dominantly function than EASM in the responsive process due to obstructed EASM by complex topography in the regions of the QTP, such as the uplift of the QTP and many mountains. The EAMS is believed to have had a tremendous influence during the Pleistocene on the genetic diversification and speciation of plants in the regions of the QTP [23,24,25,26,27]. For example, intraspecific divergence, population growth, and gene flow in two species of desert plants, *Reaumuria soongarica* and *Agriophyllum squarrosum*, appear to have been driven by the onset and stabilization of the EAMS [23,28]. Notably, the origin of *Prinsepia* species was suggested to have occurred prior to the establishment of EAMS based on a phylogenetic analysis of the Rosaceae [26]. Ma et al. reported that the divergence of *Prinsepia* species was potentially induced by the EASM, which produced a zone of humid forest vegetation during the late Oligocene [26]. This result provided an indirect evidence for the onset of a paleoclimatic event. A substantial body of data on phylogeographic patterns have provided evidence on the influence of paleoclimate events on the geographical distribution and genetic diversity of many plant species in China [7,8,29]. However, the specific impact of EAWM oscillations on population divergence and genetic diversity of species endemic to the arid northwest China has not been examined.

**Figure 1:**
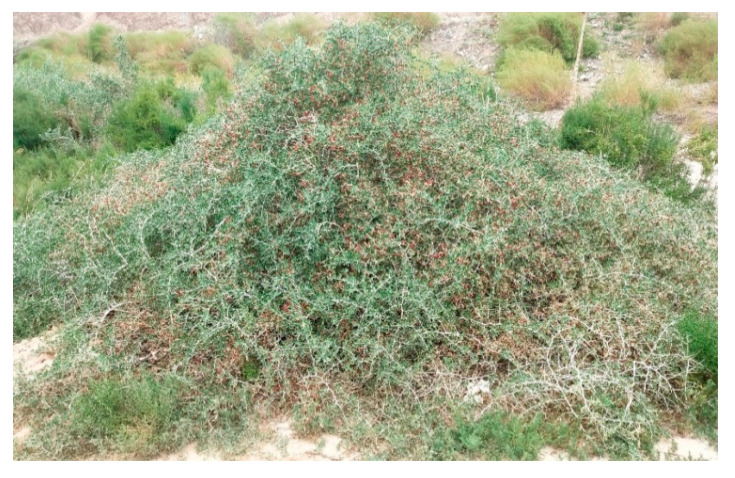
A representative plant of *Nitraria tangutorum* Bobr. investigated in this study.

*Nitraria tangutorum* Bobr., a member of the Zygophyllaceae (Figure 1), is endemic to and widely distributed in the arid region of northwest China. It is well-adapted to extremely arid environments and has substantial ecological and economic value [30]. *Nitraria tangutorum* is used to conserve soil and water, preventing desertification; a feature that plays an essential role in maintaining the ecology and climate in the regions of the QTP [31,32]. It is also a rich source of berries that possess an array of beneficial medicinal effects. Thus, *N. tangutorum* has also been used as an economic resource [30,33,34]. Notably, the geographical distribution range of *N. tangutorum* includes the EAMS fluctuation zone, which includes Kumtag, Hexi corridor, Badain Jaran, and the Tengger desert regions. Therefore, *N. tangutorum* has been proposed to be an ideal model for a better understanding of the population dynamics of arid-adapted species responding to local monsoonal climate oscillations. Therefore, in the current study, we investigated the phylogeographic pattern of *N. tangutorum* in response to EAMS using five chloroplast DNA (cpDNA) molecular markers (maternally inherited) [35]. A total of 161 individuals in 21 different populations were sampled from the East Asian monsoonal sensitive zone. The objectives of the study were: (i) to determine whether the history of regional populations of *N. tangutorum* was dynamically impacted by local monsoonal fluctuation, and ii) to identify the factors that influenced the occurrence and evolution of the phylogeographic structure of *N. tangutorum*. The results of the present study will further expand our understanding of the demographic history of drought-tolerant species (and pleiotropic benefits) in responses to the local fluctuations in monsoonal climate.

## 2. Results

### 2.1. Phylogenetic Analysis and Geographical Distribution of Haplotypes

A total of 161 *N. tangutorum* individuals, plus one sample of *N. roborowskii* as an outgroup, were collected from 21 natural localities in arid regions of northwest China (Supplementary Table S1). Five cpDNA fragments were amplified and sequenced in all the sampled individuals using the primer pairs listed in Supplementary Table S2.

**Figure 2:**
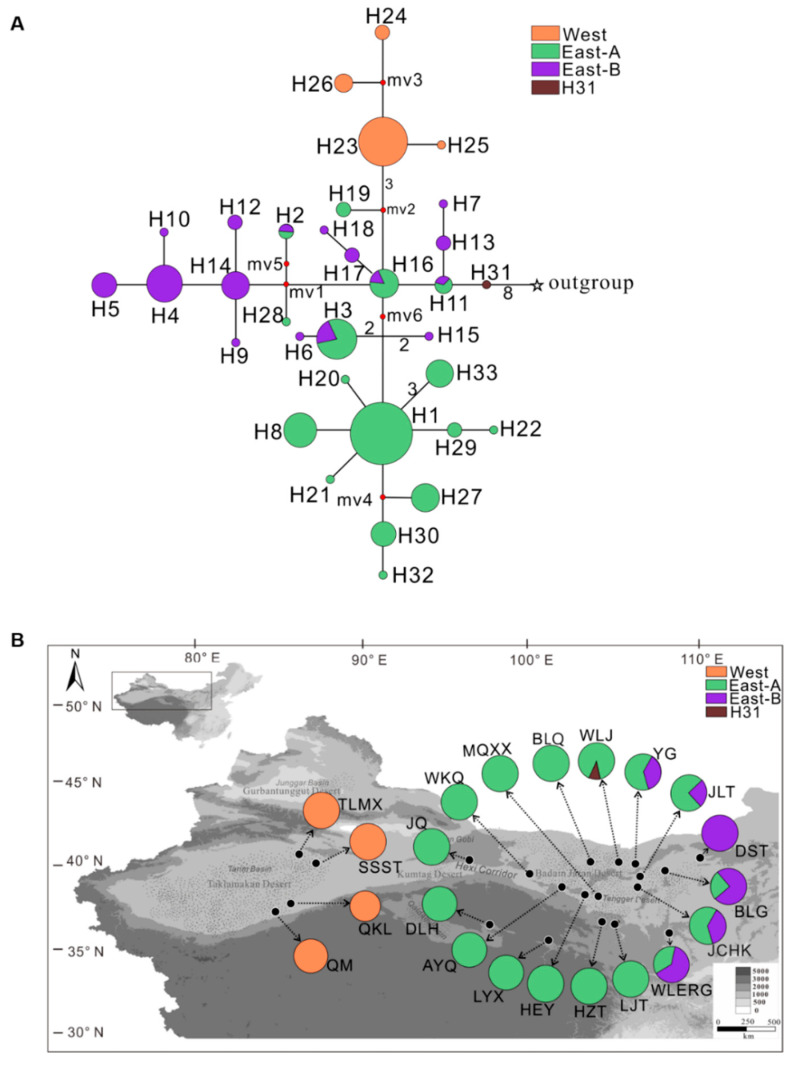
Phylogenetic topology and geographical distribution of *Nitraria tangutorum* chloroplast DNA (cpDNA) haplotypes. (**A**) Haplotype topology built using median-joining approach. Pie size is proportional to haplotype frequency, and the red circles from medium vector 1 (mv1) to mv6 indicate missing haplotypes that are not sampled in this study. Different colors of the pie diagrams denote haplotypes from different lineages. (**B**) Geographical distribution of 31 haplotypes detected in *N. tangutorum* (population codes are presented in the Table 1).

After joining the five cpDNA fragments, a 4,495-bp length of total sequence was obtained, and 33 haplotypes with 26 nucleotide substitutions were identified (Supplementary Table S3). These haplotypes clustered into three lineages based on the use of the *N. roborowskii* as an outgroup according to their phylogenetic topology (Figure 2A). The three distinct lineages were designated as West, East-A, and East-B, based on the frequency distribution of the 33 haplotypes in the population localities (Figure 2B). Among the three clades, East-A and East-B were predominantly included in the eastern area of northwest China, while the West lineage was located in the western region of China.

**Figure 3:**
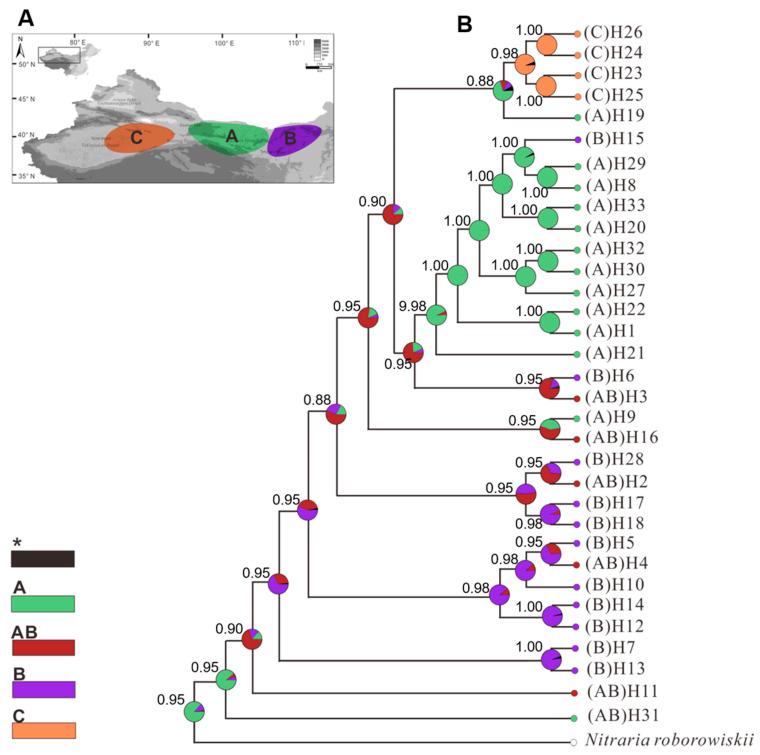
Reconstruction of the ancestral distribution of *Nitraria tangutorum* based on chloroplast DNA (cpDNA) haplotypes as determined by reconstruct ancestral state in phylogenies (RASP) analysis. (**A**) Different colors represent different origin groups as shown in the map insert. (**B**) Results of the Bayesian Binary Markov chain Monte Carlo (MCMC) analysis. The pie chart in each node indicates the possible ancestral distribution inferred from Bayesian Binary MCMC (BBM) analysis implemented in RASP. The number above the branches indicate bootstrap support values above 80. H1-H33 represent 33 haplotypes. A, East-A group; B, East-B group; C, West group; AB, East group.

Notably, the WLJ population in the East-A group contained the ancestral H31 haplotype, while the H11 haplotype, present in both the East-A and East-B lineages, was derived from the H31 haplotype (Figure 2A). These data indicate that the WLJ population may be the original center of origin of *N. tangutorum*. The phylogenetic relationship between haplotypes obtained from the reconstruct ancestral state in phylogenies (RASP) analysis supports the phylogenetic patterns obtained for *N. tangutorum* (Figure 3).

### 2.2. Population and Phylogeographical Analysis

A high degree of population divergence across the entire range of the species was revealed. The total genetic diversity, *H*_T_ (0.922, *p* = 0.034), across all populations was significantly higher than average within-population diversity, *H*_S_ (0.47, *p* = 0.081) (Table 2). Furthermore, a higher level of number of substitution types (*N*_ST_, 0.647, *p* = 0.041) than interpopulation differentiation (*G*_ST_, 0.490, *p* = 0.046), as determined by the PERMUT analysis of plastid data, indicated a significant phylogeographical structure across the species’ range (Table 2).

The analysis of molecular variance (AMOVA) indicated that the molecular variation among the three groups (51.96%) was notably higher than the variation within the populations (17.45%) (Table 3), supporting the premise that a high level of genetic differentiation exists among the three groups [*F*_CT_ = 0.520, *p* < 0.05; *Φ*_ST_ (West versus East-A) = 0.538, *p* < 0.05; *Φ*_ST_ (West versus East-B) = 0.553, *p* < 0.01; *Φ*_ST_ (East-A versus East-B) = 0.388, *p* < 0.05] (Table 3 and Table 4).

In addition, the molecular variation among populations in the East-A group (47.77%) was estimated to be slightly higher than what was present within both the East-B (43.50%) and West (33.07%) groups, suggesting that a higher degree of genetic differentiation exists in the Eastern populations compared with the West populations.

### 2.3. Estimation of the Divergence and Separation of the West and East Lineages

**Figure 4:**
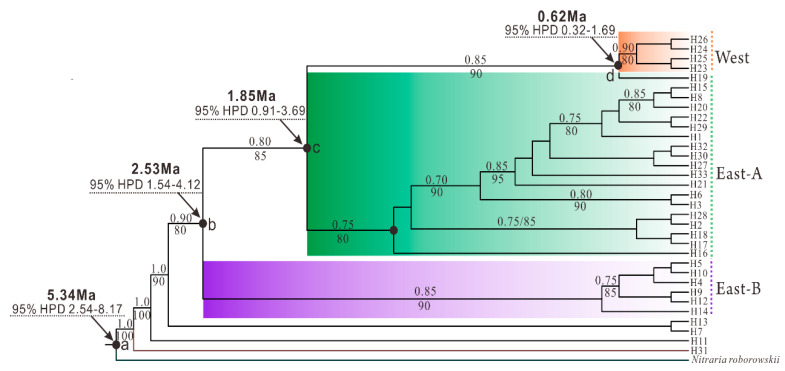
Phylogenetic relationship between *Nitraria tangutorum* haplotypes based on the concatenated dataset of five chloroplast DNA (cpDNA) fragments. Nodes indicate mean age estimates and 95% confidence intervals. Numbers above the tree branches indicate the bootstrap values calculated using posterior probabilities using BEAST software, while numbers below the tree branches represent maximum likelihood calculated using PhyML software. The black solid circles with the points “a” to “d” indicate divergence time between two clades. HPD, 95% highest posterior density.

The phylogenetic relationship constructed with all the 33 haplotypes revealed that the East-B lineage was first diverged from the ancestral haplotype H31, followed by the divergence of East-A lineage from the East-B lineage. The West lineage (4 haplotypes) was monophyletic and separated from the Eastern lineages, because it was derived the haplotype H19 that exists in the JQ population located in the eastern regions (Figure 4).

Based on the previously calculated calibrated root age as the divergence time of the outgroup species, *N. roborowskii* [36], the time of divergence between *N. tangutorum* and *N. roborowskii* could be dated to 5.34 Ma, while *N. tangutorum* became subsequently divided into East-A and East-B at 2.53 Ma as determined by the BEAST analyses (Figure 4). The time of divergence of the West lineage (0.62 Ma) from the East-A lineage was significantly posterior to divergence of the two lineages located in the eastern areas, which also suggests that the center of species origin for *N. tangutorum* was located in the eastern region of northwest China.

### 2.4. Regional Population Demography of N. tangutorum

**Figure 5:**
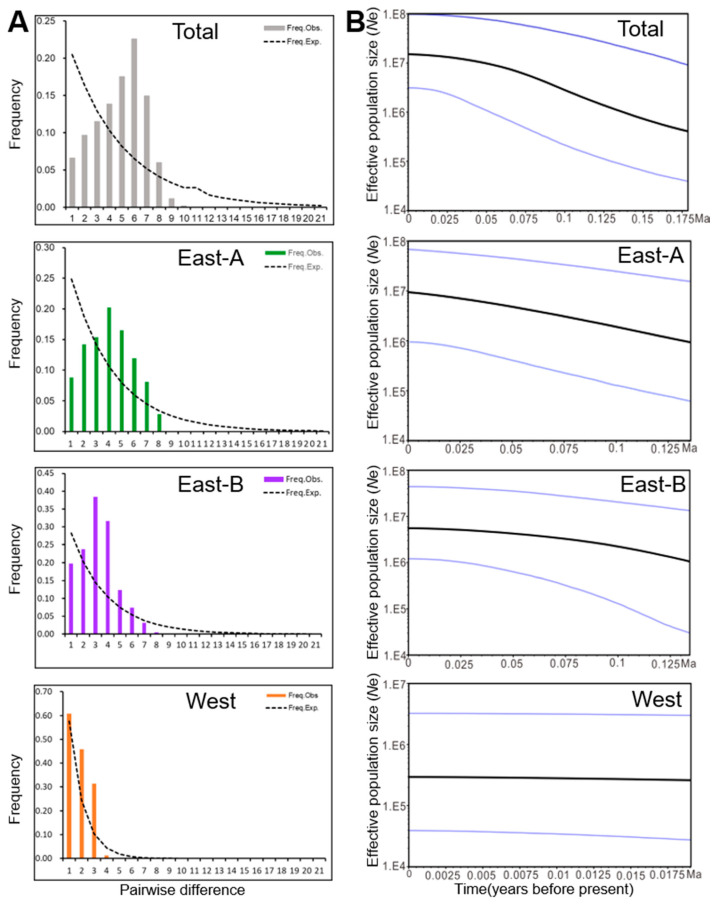
Pairwise nucleotide differences and Bayesian skyline plot (BSP) analysis for the population dynamics of *Nitraria tangutorum* based on the chloroplast DNA sequencing data. (**A**) Mismatch distribution analysis (MDA) plots for the distribution frequency of pairwise nucleotide differences in total populations or within each lineage of *N. tangutorum*. The histogram represents the observed distribution of differences among chlorotypes, while the dashed lines illustrate simulated distributions based on a model of sudden (stepwise) demographic population expansion. Freq. obs, frequency of the observed pairwise differences; Freq. exp, frequency of the expected pairwise differences; (**B**) BSP analysis of effective population sizes was determined for the entire population and individual populations of *N. tangutorum* distributed in the East-A, East-B, and West regions. The solid line indicates the median value, and the area between both blue lines represents the boundary of the 95% central posterior density interval. X-axis: time, years before present (Ma); Y-axis, effective population size (*N*e, the product of effective population size and generation length).

Obtaining neutrality tests with significantly negative values (Tajima’s *D* = −0.624, *p* = 0.034; Fu’s *Fs* = −13.614, *p* = 0.002) and the mismatch distribution analysis (MDA) results suggest that a distinct population expansion occurred in *N. tangutorum* (Table 2 and Figure 5A), which is consistent with our Bayesian skyline plot (BSP) analysis (Figure 5B). The population expansion, however, is projected to have been regional within the eastern lineages since the effective population size of the West lineage has remained relatively stable since 0.0175 Ma (Figure 5A,B).

### 2.5. Ecological Niche Modeling (ENM)

**Figure 6:**
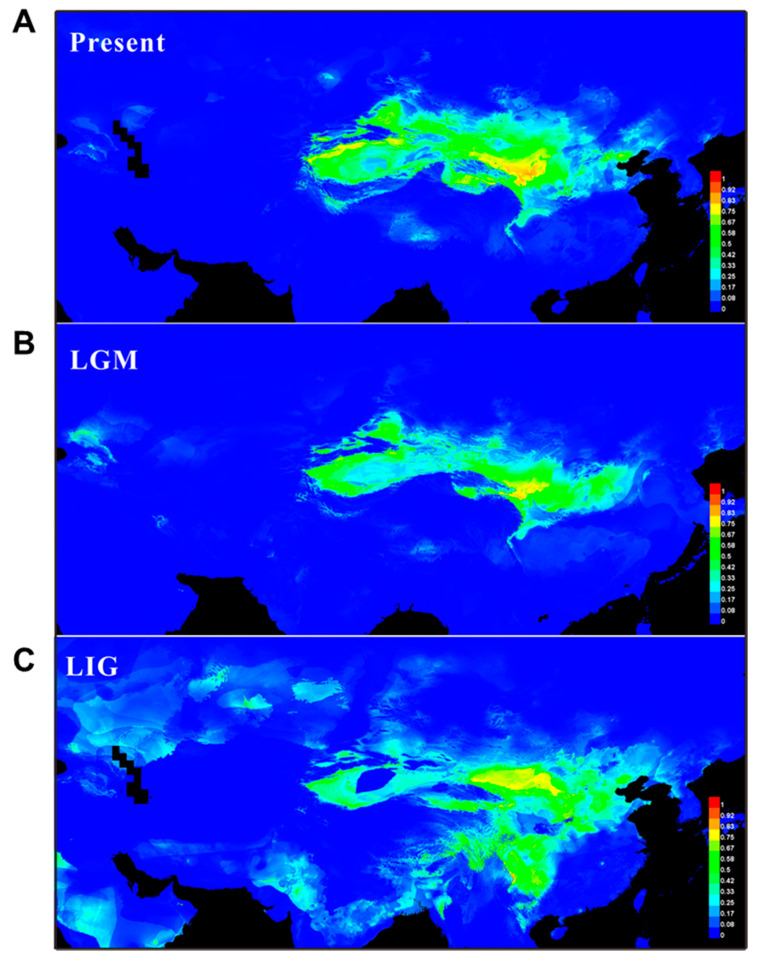
Predicted distribution of *Nitraria tangutorum* based on ecological niche modeling (ENM) using Maxent. (**A**) At the Last Inter-Glacial (LIG; ~0.14–0.12 Ma); (**B**) At the Last Glacial Maximum (LGM; ~0.021 Ma); (**C**) Under current conditions (1950–2000). The color scale in range of blue to red at the right of each image represents the lowest-to-the-highest potential distribution probability of the occurrence of *N. tangutorum*.

Based on the 206 recorded natural localities, including our sampling sites, the potential distribution of *N. tangutorum* was evaluated using Maxent software. The results revealed that the underlying niche range of *N. tangutorum* almost covers the entire eastern desert and semi-desert regions of northwest China (Figure 6A). Additionally, the area under the curve (AUC) with a value of 0.963 ± 0.014 confirmed that the ENM analysis conducted in the present study was reasonable and accurate. Furthermore, out of 19 bioclimate variants, factors Bio19 (precipitation in coldest quarter) and Bio11 (mean temperature of coldest quarter) were indicated to play a central role in determining the potential niches of *N. tangutorum* (Supplementary Table S4). The ENM data revealed that the distribution range of *N. tangutorum* fluctuated during the periods of the Last Glacial Maximum (LGM) and Last Inter-Glacial (LIG) in comparison to its present distribution. More specifically, the preferred habitats of *N. tangutorum* contracted significantly during the LGM period, while the habitats expanded and became fragmented during the LIG period (Figure 6B,C).

## 3. Discussion

### 3.1. Geographic Structure and Genetic Diversity of N. tangutorum

The impact of historical geological and paleo-climate events on the distribution and geographical structure of species has been well established in many species that inhabit the regions of the QTP [7,29,37,38]. The influence of the development and evolution of EAMS on the phylogeographic structure and genetic diversity of plant species has largely been unexplored, especially for the species located in EAMS-sensitive zones. In the present study, molecular variance analysis of cpDNA sequences was used to dissect the mechanisms underlying the population divergence and demographic history of the desert shrub species *N. tangutorum* in responses to East Asian monsoon circulation scenarios. Although 21 populations with a total of 161 individuals of *N. tangutorum* might have not covered its entire distributional ranges in the northwest China and other regions in Central Asia, these sampled populations were located in the EAMS-sensitive zones, enabling us to disentangle the above-mentioned issues.

The genetic diversity analysis (*N*st > *G*st, Table 2) displayed a significant phylogeographical structure among all populations of *N. tangutorum.* The sampled populations clustered into three distinct lineages, designated as West, East-A, and East-B lineages, based on the construction of a phylogenetic network and PhyML tree (Figure 2A and Figure 4). Notably, the haplotypes predominantly located in western and eastern regions were found to be separated into the so-called West and East lineages, respectively (Figure 2B), suggesting that molecular variance among populations is conserved and regional, as has been recently reported for several alpine species, such as *Lancea tibetica*, *Panzerina lanata*, and *Corydalis hendersonii* [6,15,39]. It is worth noting that the H31 haplotype, as a recent common ancestral haplotype, was first differentiated from the outgroup lineage with high bootstrap values, while its adjacent H11 haplotype was shared with both East-A and East-B lineages (Figure 2A and Figure 3). This finding suggests that the *N. tangutorum* population originated in the eastern region of northwest China. Notably, the phylogenetic analysis indicated that the East-A lineage diverged first (Figure 4), which is consistent with a previous report indicating that eastern central Asia is the ancestral origin of *Nitraria* [36]. Higher degrees of genetic variation were found in both East-A (*H*_T_ = 0.843) and East-B (*H*_T_ = 0.930) lineages than in the West lineage (*H*_T_ = 0.375) (Table 2), further demonstrating that population divergence in *N. tangutorum* originated in the eastern groups. Taken together, these data suggest that genetic variation is a major driver of population differentiation [40,41,42]. 

A significantly high level of pairwise genetic differentiation was evident between the western and two eastern lineages (*Φ*_ST (West versus East-A)_ = 0.538, *p* < 0.05; *Φ*_ST (West versus East-B)_ = 0.553, *p* < 0.01) (Table 4), indicating that an evident geographical isolation occurred between them. The geographic isolation may be attributed to the local mountain topography, since the complex local topography of the QTP and adjacent regions has been demonstrated to have triggered inter- or intra-species divergence, altering the phylogeographic genetic structure patterns in a variety of plant species [8,29,37,43]. The high level of genetic differentiation among the groups (*F*_CT_ = 0.520, *p* < 0.01) also indicates restricted gene flow and the high probability of genetic isolation among the groups. Similar scenarios have been found in other desert species, such as *R. soongarica* and *N. sphaerocarpa* [23,44,45]. Due to the commonality of the data obtained on the above-mentioned species, which are distributed in the arid region of northwest China [28], it is plausible to suggest that regional topography and paleoclimate have a significant impact on the adaption and evolution of the *N. tangutorum* plants. Notably, our results revealed a high-level of genetic differentiation between different populations of *N. tangutorum*, but a stable level within individual populations. These findings were made based on the fact that the level of genetic diversity among populations was approximately two-fold greater than within populations (*H*_T_ = 0.922 vs. *H*_S_ = 0.470, respectively) (Table 2). The higher differentiation level among populations of *N. tangutorum* may be due to the intensifying aridification of northwest China induced by geological and paleoclimate scenarios, such as the uplift of the QTP, the fluctuation of glacial–interglacial cycles, as well as the onset and development of EAMS [11,23,46]. We hypothesize that the regional vast deserts encompassing the range of *N. tangutorum* species, such as the Bardain Jaran and Tengger Deserts, could potentially drive population divergence by impeding gene flow between *N. tangutorum* populations.

### 3.2. Intraspecific Divergence in N. tangutorum

The highest level of genetic differentiation was observed between the eastern populations of *N. tangutorum* based on the analysis of phylogenetic structure and geographical distribution of all the identified haplotypes (Figure 2 and Figure 3). Our analysis also revealed that both East-A and East-B lineages located in the eastern region exhibited intraspecific divergence [*Φ*_ST_ (East-A versus East-B) = 0.388, *p* < 0.01], and that the East-A lineage possessed the highest level of genetic diversity (*H*_T_ = 0.930, *p* < 0.05) relative to the other lineages. An analogous phylogeographical genetic structure has been found in other xerophytes, such as *R. soongarica*, *Ephedra sinica*, *E. intermedia*, *A. squarrosum*, and *Panzerina lanata* [6,23,28,45,46]. The genetic divergence induced by the Quaternary climate oscillations was the result of this climatic event fostering habitat fragmentation, geographic isolation, and an increase in dispersal distance, which has been well established in many plant species [3,28,29,47]. The isolation of ecological niches may also contribute to genetic divergence. For example, genetic differentiation of the closely-related species *E. sinica* and *E. intermedia* resulted from changes in their ecological niches [46]. Based on the ENM analysis conducted in the present study, the potentially suitable ranges for *N. tangutorum* were significantly affected by paleoclimate changes (Figure 6). The preferred habitats of *N. tangutorum* were contracted due to the onset of a cold and dry climate during the LGM period (Figure 6B), while its habitat was extensively expanded and fragmented under the warm and moist climate of the LIG period (Figure 6C). It is widely believed that the range of a large proportion of alpine species contracted under glacial oscillations, and that this phenomenon accelerated the process of genetic divergence. This premise has been documented in many species of plants [16,19,39,48,49] and animals [50,51,52]. Two bioclimate factors, ‘precipitation’ and ‘temperature’, were greatly reduced during the LGM [53], and have been identified as the major factors determining suitable distribution ranges for *N. tangutorum* (Supplementary Table S4). These two factors are largely controlled by the EAMS, which covers the entire range of *N. tangutorum* in arid northwest China, including the Kumtag, Hexi corridor, Badain Jaran, and Tengger deserts (Figure 2B). Therefore, in addition to ecological niche changes and geographic barriers, it is apparent that the EAMS has also played a crucial role in triggering intraspecific divergence and population expansion, thus shaping the current genetic structure of the species of *N. tangutorum.*

### 3.3. Intraspecific Differentiations Triggered by the Local Monsoon Climate 

The divergence of *N. tangutorum* from *N. roborowskii* was evaluated in our study by molecular dating. Based on the variations in the cpDNA sequence, the divergence has been projected to have occurred around 5.34 Ma (Figure 4, point “a”), which is generally consistent with the results of a previous report [36]. The aridification and range fragmentation due to orogenesis also accelerated the process of genetic divergence from *N. roborowskii* as drought-tolerant *N. tangutorum* prefers arid conditions. This is a common phenomenon that has also been documented in other plant species, including *Caragana* spp. [54], *Meconopsis integrifolia* [55], *Ephedra* spp. [56], and *Phyllolobium* spp. [57]. It has also been suggested that the EASM intensified in the late Miocene–Pliocene [58,59], which may be correlated with the separation of *N. tangutorum* from *N. roborowskii*.

The intraspecific divergence of *N. tangutorum* between different populations located in the eastern region was determined to have occurred ca. 2.53 Ma (Figure 4, point “b”). During this time, the EASM decreased and the EAWM began to strengthen. These paleoclimate events, especially the EAWM and the colder and drier climate [60,61], might have facilitated intraspecific differentiation in *N. tangutorum*. We suggested that the intensification of the EAWM contributed mostly to the initial intraspecific divergence of eastern *N. tangutorum* groups (East-A and East-B lineages) based on two factors. First, it is widely believed that the EAWM resulted in a cold and dry climate that would have enhanced arid conditions in northwest China [62], environmental conditions that are representative of the preferred habitats of *N. tangutorum*. Second, the intensifying EAWM aggravated aridification and accelerated the expansion of deserts in northwest China [63], thus further fostering habitat fragmentation that could drive intraspecific divergence [38]. The EAWM steadily strengthened during the Quaternary, while the EASM underwent a gradual weakening until ~1.8 Ma [62]. Rapid diversification of eastern populations of *N. tangutorum* toward western regions occurred with the stepwise intensification of the EAWM after ~1.85 Ma (Figure 4, point “c”). The West lineage completely diverged from the East-B lineage by ~0.62 Ma (Figure 4, point “d”), a timing that could be related to EAWM-induced enhancement of habitat aridification and isolation. We propose that the EAWM fostered the dispersion of the seeds of *N. tangutorum* toward west regions, thus contributing to intraspecific divergence and allopatric differentiation in *N. tangutorum*. In addition to the establishment of the western lineage of *N. tangutorum,* further diversification of the eastern populations occurred which lead to the complete establishment of two distinct East-A and East-B lineages during the EAWM.

### 3.4. Population Demography and Colonization 

Both BSP and MDA analyses of genetic variation revealed that East-A and East-B lineages located in the EAMS zone experienced a slight population expansion (Figure 5). In particular, the potential habitat of two East lineages of *N. tangutorum* extensively expanded and fragmented during the LIG, relative to its present distribution, and then significantly contracted during the LGM. This kind of phylogeographic pattern of *N. tangutorum* appears to be consistent with the well-recognized ‘contraction/recolonization’ hypothesis described by Muellner-Riehl [10], which suggests that the species in the regions of the QTP contracted into glacial refugia during the LGM period and expanded again after the ice ages [10]. In fact, the suitable ranges of the majority of desert plants shrank during the LGM and expanded in the LIG, including species like *Larix* spp. and *E. przewalskii* [64,65]. Our findings suggest that the glacial and interglacial climates might have greatly contracted the range and accelerated the fragmentation of *N. tangutorum*, whereas the alternate climates are closely related to the East Asian monsoon. Although the influence of glacial and interglacial climates on population demography has been established in the desert plant, *Populus euphratica* [38], the impact of the monsoon system is not well understood. *Nitraria tangutorum* populations probably experienced frequent bottlenecks and habitat fragmentation during the climatic oscillations that occurred between the EAWM and EASM in the Pleistocene. It is plausible that these scenarios might have further promoted the accumulation of new mutations in local populations [38,66,67]. Consequently, this would have enlarged the effective population in the eastern climate-sensitive region as previously described [23].

**Figure 7:**
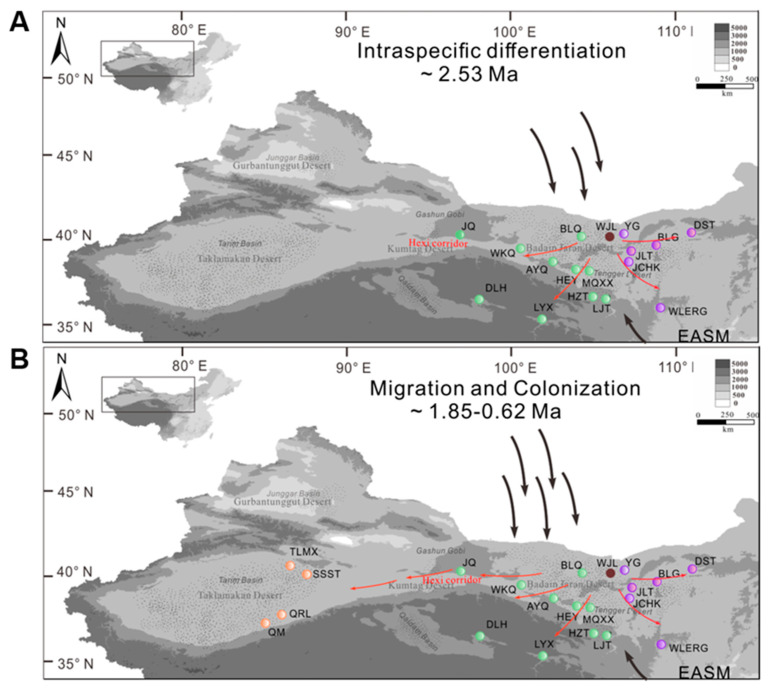
Proposed historical patterns of the migration and recolonization of *Nitraria tangutorum* in arid northwest China. (**A**) ~2.53 Ma: intraspecific differentiation occurred within the eastern groups of *N. tangutorum* triggered by the initial strengthening of the East Asian winter monsoon (EAWM). (**B**) ~1.82–0.62 Ma: putative routes of migration and recolonization of *N. tangutorum* across the Hexi corridor supported by the climatic conditions established by the EAWM. Color coding is consistent with Figure 2: brown spots represent the ancestor of *N. tangutorum*; purple, green and orange spots represent the haplotypes from the East-A, East-B, and West lineages, respectively. Red arrows represent the putative dispersal routes of *N. tangutorum*. Black arrows and number of arrows represent the direction and intensity of the EAWM and East Asian summer monsoon (EASM), respectively.

It is noteworthy that the Western lineage originated from the JQ population harboring the H19 haplotype present in the East-A lineage (Figure 2 and Table 1). This feature, along with the coincident analysis of ancestral haplotypes determined by RASP (Figure 3), strongly suggests that the western populations might have migrated from the eastern regions across the Hexi corridor and colonized the western region (Figure 7). The EAWM initially intensified during the Pliocene–Pleistocene boundary (2.6 Ma) based on numerous stratigraphic studies conducted across the Loess Plateau [60,68,69]. Thus, it appears that the East-A group expanded toward the western region, driven by the EAWM (Figure 7). Contrastingly, the surrounding mountain topography of the Hexi corridor, such as the Qilian, Longshou, Mazong, and Heli mountains, could have served as a barrier to seed dispersal in *N. tangutorum.* The Hexi corridor has been recognized as a floristic passage from central Asia to the local deserts [70], and as a possible refugium for desert plants [44,71]. Therefore, we suggest that the Hexi corridor may have served as a “dispersal corridor” for population migration of *N. tangutorum* from the eastern to the western desert regions during the intensification of the EAWM (Figure 7). 

The migration model may be common for plants in the arid northwest China, because other plant species grown in the areas adjacent to the Hexi corridor, for example, *Atraphaxis* spp., *Zygophyllum xanthoxylon*, as well as two closely related species of *N. tangutorum*, *N. roborowskii* and *N. sphaerocarpa*, are also suggested to expand toward the western regions in postglacial periods through the Hexi corridor [71,72,73]. However, the most extensive glaciation occurred in northwest China around 0.8 Ma, when the climate was extremely harsh for species survival and even led to the extinction of many plant species. Therefore, it is reasonable to suggest that the Hexi corridor was a suitable refugium for *N. tangutorum*. When the climate was milder during the last stage of glaciation, *N. tangutorum* continued to disperse toward western regions and recolonize its present locations. Subsequently, the West lineage became completely separated from the East groups around 0.62 Ma (Figure 4, point “d”) during the mid-Pleistocene. We suggest that the EAWM experienced consecutive periods of intensification after the H19 haplotype was established in the Hexi corridor region (population JQ).

## 4. Materials and Methods

### 4.1. Population Sampling

A total of 21 populations comprising 161 individuals of *N. tangutorum* were collected from its main distribution ranges in the EAMS-sensitive zone that included the Sinkiang, Gansu, Ningxia, Inner Mongolia, and Qinghai provinces (Table 1). The collection sites were all located in the arid northwest China spanning the Taklimakan desert, Qaidam basin, Kumtag desert, Hexi corridor, and Badain Jaran-Tengger desert from the west to the east. Fresh leaves were sampled from 5 to 8 individuals of each population. The sampled plants were spaced at least 30 m apart from each other to decrease sampling bias. The samples were placed in sealed envelopes and dehydrated with silica gel. The geographic location of each sampled site was recorded with a global positioning system (GPS; Garmin, Taiwan; Supplementary Table S1). *N. roborowskii* was sampled for use as an outgroup in the subsequent analyses. Voucher specimens were deposited in the Key Laboratory of Stress Physiology and Ecology in Cold and Arid Regions, Cold and Arid Regions Environmental and Engineering Research Institute, Chinese Academy of Sciences.

### 4.2. DNA Extraction, PCR Amplification and Sequencing

Genomic DNA was isolated and purified using a Plant Genomic DNA kit (Qiagen, Valencia, CA, USA), and stored at −40 °C for subsequent PCR amplification. Five pairs of cpDNA primers, *trn*H-*psb*A, *ndh*C-*trn*V, *psb*E-*pet*L, *rps*4 and *rpl*32-*trn*L were identified as being suitable for use in the study based on the prescreening of 24 primer pairs (Supplementary Table S5). The five selected primer pairs were used to generate PCR fragments of all 161 individuals of *N. tangutorum* for sequencing (Supplementary Table S2). The PCR reaction conditions and thermal programs were identical to those that were previously reported [23]. The PCR products were purified with TIAN quick Midi Purification Kits (TIANGEN, Beijing, China), and were subsequently sequenced using both forward and reverse primers on an ABI 3130xl Genetic Analyzer (Applied Biosystems, Foster City, CA, USA) platform. All the obtained sequences were examined using BioEdit v 7.0.5.3 software and aligned with ClustalX v.1.8150 with final alignment adjustments that were performed by visual inspection. All the obtained sequences of *N. tangutorum* were deposited in GenBank as accessions MT582159-MT582188.

### 4.3. Population Nucleotide Diversity and Phylogeographical Analysis

Nucleotide diversity parameters, including segregating sites (*s*), number of haplotypes (*n*), nucleotide diversity (*pi*), total haplotype diversity (*h*d), and nucleotide diversity within populations (π) in each population, were determined using DnaSP v. 5.10 software as previously described [23]. Arlequin v.3.11 software was used to generate pairwise matrices of genetic differentiation. The spatial genetic structures of chlorotypes for all *N. tangutorum* populations were determined by a spatial analysis of molecular variance by identifying the number of groups (*K*) with the highest value of genetic differentiation using SAMOVA v.1.0 software. The online tool, PERMUT (http://www.pierroton.inra.fr/genetics/lab-/software/PermutCpSSR) was employed to calculate population divergence parameters [number of substitution types (*N*_ST_) and interpopulation differentiation (*G*_ST_)], and population haplotype diversity (total-population, *H*_T_; within-population, *H*s) with 1000 random permutations. The genetic differentiation among groups (*F*_CT_), populations within group, and within populations (*F*_ST_) were determined by conducting a hierarchical analysis of molecular variance (AMOVA) using Arlequin v.3.1156 software with default parameters. The pairwise differentiations (*Φ*_ST_) between all pairs of three groups were also estimated using 1000 permutations.

### 4.4. Phylogenetic Analyses and Molecular Dating

The NETWORK v.4.6.1.2 and PhyML v.3.0 software programs were used to construct the topologies and phylogenetic relationship of chlorotypes using median-joining and maximum likelihood models, respectively. The selection of a nucleotide substitution model and ML analysis were performed as described in our previous studies [23,28]. In addition, the dating time of the divergence between lineages was evaluated by Bayesian analysis implemented using the BEAST v.1.8.0 as described in our previous study [23], except that the root age of the outgroup *N. roborowskii* lineage was set at upper 86.16 Ma and lower 57.7 Ma based on the available fossil information for *N. tangutorum* [36]. In addition, the reconstruct ancestral state in phylogenies (RASP) v.3.0 software was employed to evaluate the ancestral state and haplotype divergence using the Bayesian Binary Method (BBM) [28]. 

### 4.5. Inference of Demographic History and Ecological Niche Modelling

The predicted demographic expansion of populations was estimated through neutrality tests with Tajima’s *D*, Fu’s *F*s, and MDA using Arlequin v3.11 software. The historical dynamics of population size were also analyzed in parallel using the Arlequin v3.11 software by BSP. The preferred distributions of *N. tangutorum* were predicted based on a total of 206 recorded natural localities containing the collection sites used in this study along with online herbarium records (Chinese Virtual Herbarium, available at http://www.cvh.org.cn/). To predict the potential distribution range of *N. tangutorum* in response to glacial climatic oscillations, Maxent v.3.3 software was used to construct ecological niche modeling (ENM) under the maximum entropy algorithm using the WorldClim database from the years 1955–2000 (http://www.worldclim.org/) as described in our previous study [23].

## 5. Conclusions

The past climate oscillation and geological events have been considered as important drivers to trigger the diversification, population expansion, and speciation of plant species, especially those distributed in the QTP and adjacent regions. The evolution of EAMS, closely correlated with the multiple uplift of the QTP, has been found to result in the aridification of the central Asia interior, including the northwest China. *Nitraria tangutorum* that bears important economic and ecological values is a drought-tolerant shrub widely distributed in a broad range of sensitive zones of EAMS, especially the EAWM, in the northwest China. However, its genetic geographical patterns and demographic history under the EAWM were largely elusive. In this study, we profiled the genetic structure and population dynamic history of *N. tangutorum* using five cpDNA fragments of 161 individuals in a total of 21 populations. Three distinct lineages, including East-A, East-B, and West, were identified through the phylogenic analysis. Furthermore, the eastern regions were suggested to be the ancestral areas, wherein the split of East-A and -B lineages was significantly earlier than that of the West lineage. The higher level of genetic diversity among all populations than within populations was proposed to be closely linked to intensifying aridification of the northwest China induced by geological and paleoclimate scenarios, while the EAWM with characteristics of cold and drought enhanced the regional aridification. According to the results of ENM analysis, the potential distributions of *N. tangutorum* were predicted to contract and expand during the LGM and LIG periods, respectively, when the EAMS controlling two bioclimate factors, precipitation and temperature, predominantly influenced the surrounding environments for plant survival and led to inter- and intra-specific differentiation. Intriguingly, the West lineage was suggested to have resulted from the dispersal of *N. tangutorum* located in the eastern regions through the Hexi corridor under the strengthened EAWM during the mid-Pleistocene. Thus, EAWM was regarded as a major trigger of regional diversification and population expansion of drought-tolerant *N. tangutorum*. This study will therefore deeply facilitate our understanding of the development of EASM and its influences on phylogeographic structures and demography of drought-tolerant plants distributed in northwest China.

## Figures and Tables

**Table 1:** Geographic and genetic information for the sampled populations of *Nitraria tangutorum. s*, number of segregating sites; *h*, number of haplotypes; *H*d, estimates of haplotype diversity; π, nucleotide diversity within populations.

Population (Code)	Location (All in China)	Group	Number of Individuals	Haplotypes (Individuals)	*s*	*h*	*H*d	π
**TLMX**	Talimuxiang, Sinkiang	West	8	H23(2) H24(2) H25(1) H26(3)	4	4	0.8210	0.0004
**SSST**	Sanshisituan, Sinkiang	West	8	H23(8)	0	1	0.0000	0.0000
**QKL**	Qiongkule, Sinkiang	West	5	H23(5)	0	1	0.0000	0.0000
**QM**	Qiemo, Sinkiang	West	6	H23(6)	0	1	0.0000	0.0000
**JQ**	Jiuquan, Gansu	East-A	8	H8(3) H16(3) H19(2)	5	3	0.7500	0.0006
**WKQ**	Weikengquan, Gansu	East-A	8	H1(1) H8(1) H27(6)	4	3	0.4640	0.0003
**AYQ**	Ayouqi, IMG	East-A	8	H1(8)	0	1	0.0000	0.0000
**HEY**	Huaeryuan, Gansu	East-A	8	H1(8)	0	1	0.0000	0.0000
**MQXX**	Minqinxingxi, Gansu	East-A	8	H1(8)	0	1	0.0000	0.0000
**HZT**	Haizitan, Gansu	East-A	8	H1(4) H3(3) H11(1)	5	2	0.2500	0.0003
**LJT**	Luanjintan, Ningxia	East-A	8	H1(2) H3(2) H11(1) H20(1) H21(1) H22(1)	10	6	0.9280	0.0007
**WLJ**	Wuliji, IMG	East-A	8	H16(2) H29(1) H30(4) H31(1)	6	4	0.7500	0.0006
**BLQ**	Buliqi, IMG	East-A	8	H2(1) H3(6) H32(1)	6	3	0.4640	0.0003
**DLH**	Delingha, Qinghai	East-A	8	H8(7) H33(1)	5	2	0.2500	0.0003
**LYX**	Longyangxia, Qinghai	East-A	8	H1(8)	0	1	0.0000	0.0000
**DST**	Dashetai, IMG	East-B	7	H4(6) H10(1)	5	4	0.7140	0.0004
**WLERG**	Wulanerige, IMG	East-B	8	H3(2) H4(3) H14(2) H28(1)	6	4	0.8210	0.0005
**YG**	Yingen, IMG	East-B	8	H9(1) H4(2) H33(5)	4	4	0.6420	0.0004
**BLG**	Balagong, IMG	East-B	8	H4(2) H5(4) H6(1) H7(1)	6	4	0.7500	0.0004
**JLT**	Jilanta, IMG	East-B	8	H11(1) H14(2) H15(1) H16(1) H17(2) H18(1)	8	6	0.9280	0.0007
**JCHK**	Jichakou, IMG	East-B	7	H2(1) H3(1) H12(1) H13(2) H14(2)	8	5	0.9040	0.0006

**Table 2:** Calculation of genetic diversity and molecular variance for chlorotype sequences of *Nitraria tangutorum*. *H*_S_, average gene diversity within populations; *H*_T_, total gene diversity; *G*_ST_, interpopulation differentiation; *N*_ST_, number of substitution types; NC, not computed due to small populations; * *p* < 0.05, ** *p* < 0.01.

Regions	*H*_S_	*H*_T_	*G*_ST_	*N*_ST_	Neutrality Tests	
					Fu’s *Fs*	Tajiam’s *D*
**East-A**	0.472 (0.116)	0.843 (0.095)	0.440 (0.099)	0.691 (0.136)	−8.024 ** (0.004)	−1.306 (0.067)
**East-B**	0.824 * (0.041)	0.930 * (0.043)	0.114 * (0.022)	0.220 (NC)	−6.468 ** (0.008)	−0.077 (0.527)
**West**	0.205 * (0.020)	0.375 * (0.027)	0.452 (NC)	0.364 (NC)	−0.645 (0.359)	−1.589 * (0.046)
**Total**	0.470 (0.081)	0.922 * (0.034)	0.490 (0.046)	0.647 (0.041)	−13.614 ** 0.002	−0.624 * (0.034)

**Table 3:** Calculation of molecular variance for chlorotype sequences of *Nitraria tangutorum*. d.f., degrees of freedom; SS, sum of squares; VC, variance component; PV, percentage of variation; * *p* < 0.05.

Regions	Source of Variation	d.f.	SS	VC	PV (%)	Fixation Index
**East-A**	Among populations	10	56.705	0.623	47.770	*F*_ST_ = 0.478 *
	Within-populations	77	52.500	0.681	52.230	
	Total	87	109.205	1.305		
**East-B**	Among populations	5	38.049	0.849	43.500	*F*_ST_ = 0.435 *
	Within-populations	40	44.125	1.103	56.500	
	Total	45	82.174	1.952		
**West**	Among populations	3	3.431	0.132	33.070	*F*_ST_ = 0.331 *
	Within-populations	23	6.125	0.266	66.930	
	Total	26	9.556	0.398		
**West, East-A and East-B in comparison**	Among groups	2	121.013	1.314	51.960	*F*_CT_ = 0.520 *
	Among populations within groups	16	66.398	0.441	17.450	*F*_SC_ = 0.363 *
	Within populations	126	97.500	0.774	30.590	*F*_ST_ = 0.694 *
**Total**		144	284.910	2.530		

**Table 4:** Pairwise genetic differentiation (*Φ*_ST_) among the three lineages based on the chloroplast DNA (cpDNA) sequences of *Nitraria tangutorum.* * *p* < 0.05; ** *p* < 0.01.

	East-A	East-B	West
**East-A**	0.000 *		
**East-B**	**0.388** *	0.000 *	
**West**	**0.538** *	**0.553** **	0.000 *

## Supplementary Materials

The following are available online at https://www.mdpi.com/2223-7747/9/9/1100/s1. Supplementary Table S1. Geographical information for the sampled populations of *Nitraria tangutorum*; Supplementary Table S2. Characteristics of the primers and thermal programs for the five cpDNA fragments in this study; Supplementary Table S3. Variable sites of the aligned sequences in 33 haplotypes; Supplementary Table S4. List of the bioclimatic variables defining the ecological niche of *Nitraria tangutorum*; Supplementary Table S5. Twenty-four most variable regions in chloroplast genomes detected in this study.

## Abbreviations

QTP	Qinghai-Tibet Plateau
EAMS	East Asian monsoon system
EASM	East Asian summer monsoon
EAWM	East Asian winter monsoon
Ma	Million years ago
cpDNA	chloroplast DNA
GPS	global positioning system
AMOVA	analysis of molecular variance
ML	Maximum likelihood
RASP	reconstruct ancestral state in phylogenies
BSP	Bayesian skyline plot
MDA	mismatch distribution analysis
ENM	ecological niche modeling
LGM	Last Glacial Maximum
LIG	Last Inter-Glacial
