# Supplementary material for: The East Asian Winter Monsoon Acts as a Major Selective Factor in the Intraspecific Differentiation of Drought-Tolerant Nitraria tangutorum in Northwest China
Source: Plants (Basel). 2020 Aug 27;9(9):1100. doi: 10.3390/plants9091100 (PMC7570063; doi:10.3390/plants9091100)
Supplement: Supplementary file 1 [file plants-09-01100-s001.pdf]

Supplementary Table S1. Geographical information for the sampled populations of *Nitraria tangutorum.*

Population(code)	Location (All in China)	Latitude (N°)	Longitude (E°)	Altitude (m)
TLMX	Talimuxiang, Sinkiang	41°7.171′	83°27.101′	871.07
SSST	Sanshisituan, Sinkiang	40°40.734′	87°26.840′	848.96
QKL	Qiongkule, Sinkiang	38°31.464′	86°7.586′	1079.44
QM	Qiemo, Sinkiang	38°13.974′	85°15.882′	1202
JLT	Jilanta, IMG	39°55.637′	105°40.678′	1035.7
JCHK	Jichakou, IMG	39°31.204′	105°35.870′	1063.34
BLG	Balagong, IMG	40°15.651′	107°2.023′	1068.14
DST	Dashetai, IMG	40°56.252′	108°58.737′	1024.4
WLERG	Wulanerige, IMG	36°50.125′	107.00193	1427
YG	Yingen, IMG	40°49.815′	105°11.239′	1211.38
JQ	Jiuquan,,Gansu	40°47.886′	96°7.069′	1339
WKQ	Weikengquan, Gansu	40°16.713′	99°49.117′	1138.32
AYQ	Ayouqi, IMG	39°15.065′	101°40.508′	1517.56
HEY	Huaeryuan, Gansu	38°54.833′	102°21.508′	1338.27
MQXX	Minqinxingxi, Gansu	38°53.584′	103°20.310′	2639
HZT	Haizitan, Gansu	37°26.137′	103°55.303′	1798.74
LJT	Luanjintan, Ningxia	41°42.156′	105°22.178	1427
WLJ	Wuliji, IMG	40°38.753′	107°8.196′	1252.23
BLQ	Buliqi, IMG	41°15.651′	107°2.023′	1683.62
DLH	Delingha, Qinghai	37°18.824′	97°23.030′	1373
LYX	Longyangxia, Qinghai	36° 7.952′	100°41.188′	1201

Supplementary Table S2. Characteristics of the primers and thermal programs for the five chloroplast DNA (cpDNA) fragments used in this study.

Region	Primer （5′→3′）	Sequence	Sequence length(bp)	TM (℃)	Reference
*trn*H-*psb*A	*trn*H-F<br>*psb*A-R	CGCGCATGGTGGATTCACAAATC<br>TGCATGGTTCCTTGGTAACTTC	680	60	[1]
*ndh*C-*trn*V	*ndh*C-F<br>*trn*V-R	AGACCATTCCAATGCCCCCTTTCGCC<br>GTTCGAGTCCGTATAGCCCTA	680	60	[2]
*psb*E--*pet*L	*psb*E-F<br>*pet*L-R	AACAAAAGGATTCGCAAATAAAAG<br>AGTTGTTGTTCTTGTTTCTTTAGT	680	60	[1]
*rpl*32-*trn*L	*rpl*32-F<br>*trn*L-R	GCGTATTCGTAAAAATATTTGGAA<br>TTCCTAAGAGCAGCGTGTCTACC	870	53	[1]
*rps*4	*rps*4-F<br>*rps*4-R	ATGTCCCGTTATCGAGGACCT<br>TACCGAGGGTTCGAATC	730	52	[1]

References

1. Dong, W.; Liu, J.; Yu, J.; Wang, L.; Zhou, S., Highly Variable Chloroplast Markers or Evaluating Plant Phylogeny at Low Taxonomic Levels and for DNA Barcoding. *PloS one*, **2012**, *7*, e35071.
2. Qian, C.; Yin, H.; Shi, Y.; Zhao, J.; Yin, C.; Luo, W.; Dong, Z.; Chen, G.; Yan, X.; Wang, X. R.; Ma, X. F., Population dynamics of *Agriophyllum squarrosum*, a pioneer annual plant endemic to mobile sand dunes, in response to global climate change. *Sci. Rep*. **2016**, *6*, 26613.

Supplementary Table S3. Variable sites of the aligned sequences in 33 haplotypes.

Haplotype	Nucleotide variable positions																									
	*ndh*C-*trn*V			*trn*H-*psb*A										*psb*E-*pet*L					*rps*4			*rpl*32-*trn*L				
	297	461	604	725	986	1534	1538	1552	1598	1626	1685	1746	1751	1757	1758	1761	1762	1795	2455	2654	2701	2724	2731	2736	3939	4071
H1	T	A	A	T	G	A	G	G	T	A	T	T	A	G	A	G	A	T	A	G	G	T	T	T	G	A
H2	T	A	A	T	G	A	G	T	T	A	T	T	A	G	G	G	A	T	A	G	A	G	T	T	G	A
H3	T	A	A	T	G	A	G	G	T	A	T	T	A	G	G	G	A	T	A	G	A	T	T	T	A	A
H4	T	A	A	T	G	A	G	G	T	A	T	T	G	G	G	A	A	T	C	G	A	T	T	T	A	A
H5	T	A	A	T	G	A	G	G	T	A	T	T	G	G	A	A	A	T	C	G	A	T	T	T	A	A
H6	T	A	A	T	G	A	G	G	T	A	T	T	A	G	G	G	A	T	C	G	A	T	T	T	A	A
H7	T	A	A	T	G	A	G	G	T	A	T	T	G	G	G	A	A	A	A	G	G	T	T	T	A	A
H8	T	A	A	T	G	A	G	G	T	A	T	T	A	G	G	G	A	T	A	G	G	T	T	G	G	A
H9	T	A	A	T	G	A	G	G	G	A	T	T	G	G	G	A	A	T	A	G	A	T	T	T	G	A
H10	T	A	A	T	G	A	G	G	T	A	T	T	G	G	G	A	A	T	C	G	G	T	T	T	A	A
H11	T	A	A	T	G	A	G	G	T	A	T	T	G	G	G	A	A	A	A	G	G	T	T	T	G	A
H12	T	A	A	T	G	A	G	G	T	A	G	A	G	G	G	A	A	T	A	G	A	T	T	T	A	A
H13	T	A	A	T	G	A	G	G	T	A	T	T	G	G	G	A	A	A	A	G	A	T	T	T	A	A
H14	T	A	A	T	G	A	G	G	T	A	T	T	G	G	G	A	A	T	A	G	A	T	T	T	A	A
H15	T	A	A	T	G	A	G	G	T	C	T	T	A	G	G	G	A	T	A	G	G	T	T	T	G	A
H16	T	A	A	T	G	A	G	G	T	A	T	T	G	G	G	A	A	T	A	G	G	T	T	T	G	A
H17	T	C	A	T	G	A	G	T	T	A	T	T	G	G	G	A	A	T	A	G	G	T	T	T	G	A
H18	T	A	A	T	G	A	G	T	T	A	T	T	G	G	G	A	A	T	A	G	G	T	T	T	G	A
H19	T	A	T	T	A	A	G	G	T	A	T	T	G	G	G	A	A	T	A	G	G	T	T	T	G	A
H20	T	A	A	T	G	A	G	G	T	A	G	T	A	G	G	G	A	T	A	G	G	T	T	T	G	A
H21	T	A	A	T	G	A	G	G	T	A	T	T	A	C	G	G	G	A	A	G	G	T	T	T	G	A
H22	T	A	A	T	G	T	G	G	T	A	T	T	A	C	A	G	A	T	A	G	G	T	T	T	G	A
H23	T	A	T	A	G	A	G	G	T	A	T	T	G	G	G	A	A	T	A	G	G	T	T	T	G	A
H24	T	A	T	A	G	A	G	G	T	A	T	T	G	C	G	A	A	T	A	G	A	T	T	T	A	A
H25	T	A	T	A	G	A	G	G	T	A	T	T	G	G	G	A	A	T	A	G	G	T	G	T	G	A
H26	T	A	T	A	G	A	G	G	T	A	T	T	G	C	G	A	A	T	A	G	A	G	T	T	G	A
H27	T	A	A	T	G	A	G	G	T	A	T	T	A	G	G	G	A	T	A	A	G	T	T	T	G	T
H28	T	A	A	T	G	A	G	T	T	A	T	T	G	G	G	A	A	T	A	G	A	G	T	T	G	A
H29	T	A	A	T	G	A	G	G	T	A	T	T	A	G	G	G	A	T	A	G	G	T	T	T	G	A
H30	T	A	A	T	G	A	C	G	T	A	T	T	A	G	G	G	A	T	A	G	G	T	T	T	G	T
H31	T	A	A	T	G	A	G	G	T	A	T	T	G	G	G	A	A	A	A	G	G	T	T	T	G	A
H32	G	A	A	T	G	A	C	G	T	A	T	T	A	G	G	G	A	T	A	G	G	T	T	T	G	T
H33	T	A	A	T	G	A	G	G	T	A	T	T	A	G	G	G	A	T	A	G	G	T	T	T	G	A

Supplementary Table S4. Estimated results of relative contributions of the bioclimatic variables to the ecological niche of *Nitraria tangutorum*.

code	Bioclimatic variables	Percent contribution	Permutation importance
BIO19	Precipitation in Coldest Quarter	**41.7**	**49.1**
BIO11	Mean Temperature of Coldest Quarter	**24.6**	**30.5**
BIO18	Precipitation in Warmest Quarter	8.8	4.4
BIO9	Mean Temperature of Driest Quarter	5.8	3.8
BIO14	Precipitation in Driest Month	4.3	2.9
BIO4	Temperature Seasonality	3.1	2.4
BIO13	Precipitation in Wettest Month	2.9	1.7
BIO5	Max Temperature of Warmest Month	2.6	1.5
BIO8	Mean Temperature of Wettest Quarter	2.1	1.3
BIO2	Mean Diurnal Range	2.0	0.9
BIO3	Isothermality	1.9	1.1
BIO1	Annual Mean Temperature	0.2	0.4

Note: Significant contributions over 10% are indicated in bold text.

Supplementary Table S5 Twenty-four most variable regions in chloroplast genomes detected in this study.

Code	Region	name	Forward	Name	Reverse
**1**	*pet*B-*pet*D	*pet*B-F	CAATCCACTTTGACTCGTTTT	*pet*D-R	GGTTCACCAATCATTGATGGTTC
**2**	*clp*P	*clp*P-F	GCTTGGGCTTCTCTTGCTGACAT	*clp*P-R	TCCTAATCAACCGACTTTATCGAG
**3**	*atp*H-*atp*I	*atp*H-F	AACAAAAGGATTCGCAAATAAAAG	*atp*I-R	AGTTGTTGTTCTTGTTTCTTTAGT
**4**	*trn*H-*psb*A	*Trn*H-F	CGCGCATGGTGGATTCACAAATC	*Psb*A-R	TGCATGGTTCCTTGGTAACTTC
**5**	*trn*T-*psb*D	*trn*T-F	GCCCTTTTAACTCAGTGGTAGAG	*psb*D-R	CCAAATAGGAACTGGCCAATC
**6**	*acc*D-*psa*I	*acc*D-F	GGTAAAAGAGTAATTGAACAAAC	*psa*I-R	GGAAATACTAAGCCCACTAAAGGCAC
**7**	*trn*S2-[illegible]	*trn*S2-F	CGGTTTTCAAGACCGGAGCTATCAA	*trn*G2-R	CATAACCTTGAGGTCACGGGTTCAAAT
**8**	*atp*H-*atp*I	*atp*H-F	AACAAAAGGATTCGCAAATAAAAG	*atp*I-R	AGTTGTTGTTCTTGTTTCTTTAGT
**9**	*psb*M-*trn*D	*psb*M-F	TTTGACTGACTGTTTTTACGTA	*trn*D-R	CAGAGCACCGCCCTGTCAAG
**10**	*ndh*C-*trn*V	*ndh*C-F	AGACCATTCCAATGCCCCCTTTCGCC	*trn*V-R	GTTCGAGTCCGTATAGCCCTA
**11**	*ndh*F	*ndh*F-F	ACACCAACGCCATTCGTAATGCCATC	*Ndh*F-R	AAGATGAAATTCTTAATGATAGTTGG
**12**	*pet*A-*psb*J	*pet*A-F	GGATTTGGTCAGGGAGATGC	*psb*J-R	ATGGCCGATACTACTGGAAGG
**13**	*psb*E--*pet*L	*psb*E-F	ATCTACTAAATTCATCGAGTTGTTCC	*pet*L-R	TATCTTGCTCAGACCAATAAATAGA
**14**	*ndh*A	*Ndh*A-[illegible]	TCAACTATATCAACTGTACTTGAAC	*Ndh*A-R	CGAGCTGCTGCTCAATCGAT
**15**	*rbc*L-*acc*D	*rbc*L-F	TAGCTGCTGCTTGTGAGGTATGGA	*Acc*D-R	AAATACTAGGCCCACTAAAGG
**16**	*rpl*32-*trn*L	*rpl*32-F	GCGTATTCGTAAAAATATTTGGAA	*trn*L-R	TTCCTAAGAGCAGCGTGTCTACC
**17**	*rpo*B-*trn*C	*rpo*B-F	ACAAAATCCTTCAAATTGTATCTGA	*trn*C-R	TTTGTTAATCAGGCGACACCCGG
**18**	*rps*16-*trn*Q	*rps*16-F	TTTATCGGATCATAAAAACCCACT	*trn*Q-R	TGGGGCGTGGCCAAGCGGT
**19**	*pet*N-*psb*M	*pet*N-F	ATGGATATAGTAAGTCTCGCTTGG	*psb*M-R	ATGGAAGTAAATATTCTTGCAT
**20**	*rps*4	*rps*4-F	ATGTCCCGTTATCGAGGACCT	*rps4*-R	TACCGAGGGTTCGAATC
**21**	*trn*K	*trn*K-F	GGGACTCGAACCCGGAACTA	*trn*K-R	AGTACTCGGCTTTTAAGTGCG
**22**	*trn*S^{GCU}-[illegible]	*trn*S^{GCU}	AACGGATTAGCAATCCGACGCTTTA	*trn*G^{GCC}	CTTTTACCACTAAACTATACCCGC
**23**	*trn*S^{UGA}-[illegible]	*trn*S^{UGA}	CGGTTTTCAAGACCGGAGCTATCAA	*trn*G^{UCC}	CATAACCTTGAGGTCACGGGTTCAAAT
**24**	*trn*W-*psa*J	*trn*W-F	TCTACCGAACTGAACTAAGAGCGC	*psa*J-R	CGATTAATCTCTATCAATAGACCTGC
